# The structural, dynamic, and thermodynamic basis of darunavir resistance of a heavily mutated HIV-1 protease using molecular dynamics simulation

**DOI:** 10.3389/fmolb.2022.927373

**Published:** 2022-08-15

**Authors:** Yaser Shabanpour, Sharareh Sajjadi, Esmaeil Behmard, Parviz Abdolmaleki, Amir Homayoun Keihan

**Affiliations:** ^1^ Molecular Biology Research Center, Systems Biology and Poisonings Institute, Baqiyatallah University of Medical Sciences, Tehran, Iran; ^2^ Department of Biology, Roudehen Branch, Islamic Azad University, Roudehen, Iran; ^3^ Pharmaceutical Sciences Research Center, Shiraz University of Medical Sciences, Shiraz, Iran; ^4^ Department of Biophysics, Faculty of Biological Sciences, Tarbiat Modares University, Tehran, Iran

**Keywords:** HIV-1 protease, darunavir, molecular dynamics simulation, drug resistance, PCA analysis

## Abstract

The human immunodeficiency virus type 1 protease (HIV-1 PR) is an important enzyme in the life cycle of the HIV virus. It cleaves inactive pre-proteins of the virus and changes them into active proteins. Darunavir (DRV) suppresses the wild-type HIV-1 PR (WT-Pr) activity but cannot inhibit some mutant resistant forms (MUT-Pr). Increasing knowledge about the resistance mechanism can be helpful for designing more effective inhibitors. In this study, the mechanism of resistance of a highly MUT-Pr strain against DRV was investigated. For this purpose, complexes of DRV with WT-Pr (WT-Pr-D) and MUT-Pr (MUT-Pr-D) were studied by all-atom molecular dynamics simulation in order to extract the dynamic and energetic properties. Our data revealed that mutations increased the flap-tip flexibility due to the reduction of the flap-flap hydrophobic interactions. So, the protease’s conformation changed from a closed state to a semi-open state that can facilitate the disjunction of DRV from the active site. On the other hand, energy analysis limited to the final basins of the energy landscape indicated that the entropy of binding of DRV to MUT-Pr was more favorable than that of WT-Pr. However, the enthalpy penalty overcomes it and makes binding more unfavorable relative to the WT-Pr. The unfavorable interaction of DRV with R8, I50, I84, D25′, and A28′ residues in MUT-Pr-D relative to WT-Pr-D is the reason for this enthalpy penalty. Thus, mutations drive resistance to DRV. The hydrogen bond analysis showed that compared with WT-Pr, the hydrogen bonds between DRV and the active-site residues of MUT-Pr were disrupted.

## 1 Introduction

The human immunodeficiency virus type 1 protease (HIV-1 PR) is an enzyme that cleaves the HIV polyproteins (the Gag and Gag-pol), helping the virus to reach maturity at the last stage of its life cycle (Sillapachaiyaporn and Chuchawankul, 2020). So, the enzyme inhibitors may play an important role in the battle with acquired immunodeficiency syndrome (AIDS) disease (Ghosh et al., 2016). However, the strains with a mutant protease demonstrate a great resistance against inhibitors (Wang et al., 2020). Therefore, understanding the molecular mechanisms underlying the resistance of mutant HIV-1 PR toward antiviral drugs is crucial for the design of highly potent inhibitors against drug-resistant strains.

HIV-1 PR is a member of the aspartyl protease family, which contains the aspartate residue in its active site (Mager, 2001). This protein is a symmetric homodimer that has two identical chains (i.e., A and B), each containing 99 residues (Sahin, 2020). The D25, T26, G27 (chain-A), D25’, T26’, and G27’ (chain-B) residues from the catalytic site (Mager, 2001). HIV-1 PR has six main structural segments, namely fulcrum (A and B: 11–22, 11’–22’), active site (A and B: 23–30, 23’–30’), flap-elbow (A and B: 35–42, 35’–42’), flap-tip (A and B: 43–58, 43’–58’), cantilever (A and B: 59–75, 59’–75’), and interface (A and B: 95–99, 95’–99’) (Supplementary Figure S1A) (Yu et al., 2017). The flap-tips are glycine-rich domains with a hairpin structure, which control the access of substrate/inhibitor to the active site (Tie et al., 2004). It has been shown both computationally and experimentally that the HIV-1 PR has three possible conformations, namely closed, semi-open, and open-like (i.e., curled, open, and wide open) conformations, which are classified based on the distance between two flap-tips (Huang et al., 2017; Apoorva and Sasidhar, 2020; Badaya and Sasidhar, 2020; Liu et al., 2020; Sk et al., 2020). In the ligand-bound form, the flap-tips take a downward conformation relative to the active site (closed state), while the free form permanently takes a semi-open conformation (Hornak et al., 2006; Hornak and Simmerling, 2007). The inhibitor pressure selected mutations to lead to forming open-like conformations and destabilize the closed conformation (Carter et al., 2014b; Huang et al., 2014; Liu et al., 2016; Liu et al., 2020). It has been discovered that there is a linear correlation between DRV inhibition and the population ratio of open-like to the closed state (Liu et al., 2020). The opening of the flap-tips is presumably essential to allow the entry of substrate to the active site but this conformation was not detected by the crystallographic experiments due to its short lifetime (Lapatto et al., 1989; Navia et al., 1989). However, the nuclear magnetic resonance (NMR) experiments could show the flexibility of the flap-tips, which undergo sub-nanosecond time scale fluctuations (Roche et al., 2015; Deshmukh et al., 2017). According to the NMR data, all of the three possible conformations are in dynamic equilibrium, whereas the semi-open state is dominant in the ligand-free HIV-1 PR (Ishima et al., 1999; Freedberg et al., 2002; Katoh et al., 2003). This hypothesis was further approved by the molecular dynamic (MD) simulation studies (Scott and Schiffer, 2000; Tozzini and McCammon, 2005; Hornak et al., 2006; Gupta and Senapati, 2019; Apoorva and Sasidhar, 2020).

Darunavir (DRV) is one of the HIV-1 PR inhibitors, which belongs to the second generation of AIDS drugs, with very limited side effects, approved by the FDA^1^, (Darwish et al., 2020). It has been shown that the oxygen atoms of the bis-tetrahydrofuran (bis-THF) group of DRV (Supplementary Figure S1B) can interact with the backbone and side-chain atoms of D30 and D30’ residues of HIV-1 PR by forming hydrogen bonds (HBs) (Kovalevsky et al., 2006; Orkin et al., 2020). DRV is a non-peptide compound that is proven to have potent activity against drug-resistant HIV-1 strains (Ghosh et al., 2007; Kovalevsky et al., 2008). So, it has been widely used in AIDS treatment (Chow et al., 2020; Navarro et al., 2020; Santos et al., 2020). However, there are some mutations that lead to resistance of the HIV-1 PR to DRV. The main substituted residues include V11I, V32I, L33F, I47V, I50V, I54M, G73T, T74P, L76V, I84V, and L89V, among which the most prevalent ones were I47V, I54M, I84V, and G73T substitutions (Johnson et al., 2008; Tremblay, 2008).

The use of drugs against the HIV-1 PR increases the diversity of its mutant strains (Carter et al., 2014a). Recently, ∼50 mutations have been discovered at 30 different sites of the HIV-1 PR gene (Wensing et al., 2019). The mutations fall into two types: 1) mutations that occur in the active site, which directly reduce the drug-protease interactions (proximal mutations); 2) mutations that occur distant from the active site (distal mutations), which indirectly reduce the HIV-1 PR affinity for the drug by affecting the conformational dynamics of the enzyme (Johnson et al., 2008; Meher and Wang, 2012). However, some mutations may exert both effects.

This study was devoted to comparing the structural, dynamic, and thermodynamic features of MUT-Pr-D (PDB code:3TTP) and WT-Pr-D by using MD simulation in atomistic details. This mutant strain was derived from clinical samples harboring mutations associated with high DRV resistance (Šašková et al., 2009). Although the study of this mutant strain is an important issue, to the best of our knowledge, it has not yet been studied. The substituted residues of this mutant enzyme were I13V, K20R, V32I, L33F, E35D, M36I, S37N, R41K, K43T, I47V, I54M, I62V, L63V, A71V, I72T, G73S, T74P, V82L, L89V, and I93L (Kožíšek et al., 2014), which, as stated before, comprises the most prevalent mutations that lead to resistance against DRV in AIDS patients. Our data revealed that mutations could increase the flexibility of the flap-tips, make them separated relative to each other, and change the motion of the fulcrums, cantilevers, and flap-elbows, which helps the conversion of the protease’s conformation from a closed state to a semi-open state. This consequently facilitates the disjunction of DRV from the active site. Besides, the more unfavorable binding enthalpy in MUT-Pr-D relative to WT-Pr-D overcomes the more favorable binding entropy, which leads to protease resistance against DRV. Furthermore, it was found that hydrogen bonds between DRV and the active site residues of MUT-Pr were less than those of WT-Pr. The mechanistic knowledge from this study may provide clues for designing new inhibitors against the HIV-1 protease.

## 2 Materials and methods

### 2.1 System preparation

The 3-dimensional structures of WT-Pr-D (PDB:1T3R) (Surleraux et al., 2005) and MUT-Pr-D (PDB:3TTP) (Kožíšek et al., 2014) were obtained from the protein data bank (PDB). Missing atoms were added to the structures using the Swiss-Pdb viewer (Guex and Peitsch, 1997). Due to the importance of the protonation state of D25’, one proton was added to its oxygen atom (OD2) in both WT-Pr-D and MUT-Pr-D (Hyland et al., 1991; Wang et al., 1996). The charges of the DRV were obtained by using the restrained electrostatic potential (RESP) method (Bayly et al., 1993). The general amber force field (GAFF) (Wang et al., 2004) parameters and the RESP charges were determined for DRV by using the antechamber module of the AMBER20 package (Case et al., 2020). All missing hydrogen atoms were added using the LeaP module. The WT-Pr-D and MUT-Pr-D were solvated with the TIP3P (Jorgensen et al., 1983) water model in the periodic boxes of sizes 7.71, 7.71, and 5.45 (x, y, and z: nm) and 8.14, 8.14, and 5.75 (nm), respectively. The chloride ions were added in order to neutralize the positive charges of the systems. In the WT-Pr-D system, 9697 TIP3P water molecules and 7 Cl ions, and in the MUT-Pr-D system, 11533 water molecules and 3 Cl ions were added to the simulation boxes. Then, the topology configurations for both systems were built by GROMACS version 2019 and the ff99SB force field (Koes and Vries, 2017).

### 2.2 Molecular dynamics simulation

In the first step, the WT-Pr-D and MUT-Pr-D, in water boxes, were minimized by using the steepest descent minimization algorithm for 150000 steps. Constant temperature and pressure conditions were applied for both simulations (Ryckaert et al., 1977; Berendsen et al., 1984). The systems were equilibrated at the NVT ensemble for 150,000 picoseconds (ps) by using a leap-frog integrator. In all simulations, the time step was set as 2 femtoseconds (fs) and the temperature was coupled *via* the V-rescale algorithm. At last, the temperature of systems was equilibrated at 310 K and followed by the NPT ensemble equilibration for another 150,000 ps. The pressure coupling and its type were determined to be Berendsen and isotropic. The pressure of the systems was equilibrated at 1 bar.

For all NVT ensembles, NPT ensembles, and MD production, the grid-based neighbor list update was used with 1.2 nm as a cut-off. The cut-off of Lennard-Jones interactions was determined as 1.2 nm. Long-range electrostatic interactions were calculated by the particle mesh ewald (PME) method (Essmann et al., 1995). The cut-off of the short-range electrostatic interactions was determined as 1.2 nm. At last, the 200 ns production simulations for WT-Pr-D and MUT-Pr-D systems were performed. This time was chosen based on the timescale for studying the protein domain motions (Cai et al., 2012; Ode et al., 2012; Rana et al., 2022) and the time needed to reach stable simulating structures, in which the average RMSD reached straight lines.

### 2.3 Principal component analysis method

The principal component analysis (PCA) method (David and Jacobs, 2014) is often used to extract the main dynamical properties of the MD trajectories (Bahar et al., 1998). In order to perform the PCA analysis, the gmx covar tool of GROMACS was used to extract the covariance matrix, eigenvectors, and eigenvalues from 20,000 frames of two system trajectories during a 200 ns simulation.

The eigenvectors represent the direction of motion and the eigenvalues represent the mean square fluctuation in these directions (Du et al., 2006; David and Jacobs, 2014). The principal components (PCs) were obtained by diagonalization of the covariance matrix and calculation of the eigenvalues and eigenvectors (David and Jacobs, 2014). At last, the gmx anaeig tool was used for the projection of trajectories onto the eigenvectors to give the PCs. The first five PCs commonly describe >75 percent of the system’s global motions, in which the first PC contains the largest movements in the ensemble (Maisuradze et al., 2009). All conformations of MUT-Pr, WT-Pr, and DRV in complexes during 200 ns were separately applied to PCA analysis. The free energy landscape, the binding entropy, and essential dynamics for both systems were obtained from PCA analysis.

### 2.4 Molecular mechanics-poisson boltzman surface area approach

The molecular mechanics-poisson Boltzmann surface area (MM-PBSA) approach was used to calculate the binding enthalpy between HIV-1 proteases and DRV in both systems. For this purpose, the python script MM-PBSA.py (Kumari et al., 2014) was used to calculate the average binding energies in the last basins, which were extracted *via* PCA analysis from both trajectories.

## 3 Results and discussion

### 3.1 Simulation stability

The root mean square deviation (RMSD) for the backbone atoms of the proteases and heavy atoms of DRV in MUT-Pr-D and WT-Pr-D were analyzed to find out if the structures reached stable states during the simulations (Supplementary Figures S2A and B). The mean RMSD and SD for the backbone atoms were calculated to be 0.12 ± 0.01 nm and 0.11 ± 0.01 nm for the proteases in WT-Pr-D and MUT-Pr-D, respectively. So, both the mutant and wild-type proteases reached their stable states at the RMSD value of almost 0.1 nm (Supplementary Figure S2A). The mean RMSD was slightly higher (0.01 nm) in protease of WT-Pr-D with respect to MUT-Pr-D. As seen in Supplementary Figure S2B, the DRV in MUT-Pr-D showed more structural deviations until 110 ns and then reached a steady state at a mean RMSD of 0.12 ± 0.01 nm. The DRV in WT-Pr-D complex reached a stable state sooner than that in MUT-Pr-D at a RMSD of 0.12 ± 0.01 nm. It also showed lower structural deviations at earlier steps of simulation (Supplementary Figure S2B). These data indicated that in both the WT-Pr-D and MUT-Pr-D complexes, DRV reached its stable state during the simulation.

### 3.2 Comparison of the flexibility of the residues

**FIGURE 1:**
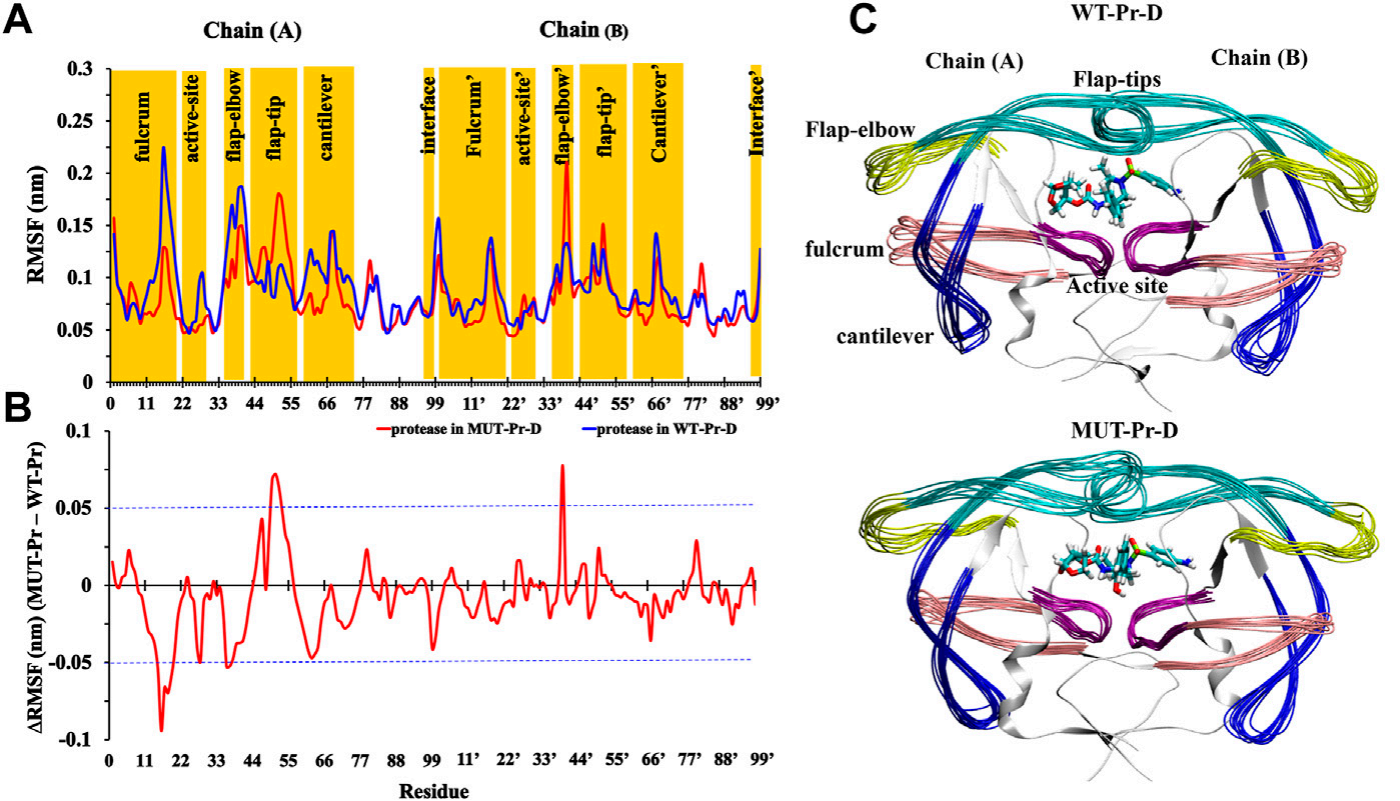
**(A)** RMSF values of C_α_ atoms of WT-Pr-D (blue) and MUT-Pr-D (red); **(B)** the difference in RMSF values between MUT-Pr-D and WT-Pr-D were illustrated and residues with an absolute difference larger than 0.05 nm (exceeding the blue cutoff lines) were considered as highly fluctuated; **(C)** 10 superimposed trajectories snapshots of WT-Pr-D and MUT-Pr-D with 2 ns intervals were illustrated.

In order to evaluate the effect of mutations on the residual flexibility, the root mean square fluctuations (RMSF) of the C_α_ atoms were calculated for the proteases in MUT-Pr-D and WT-Pr-D complexes (Figure 1A), and the RMSF difference is shown in Figure 1B. The residues with an RMSF difference of more than 0.05 nm were considered to be highly fluctuating, which represents significant mutation-induced conformational changes. As seen, the residues D25-T26-G27 and D25’-T26’-G27’ of the catalytic site indicated low flexibility in both the WT-Pr-D and MUT-Pr-D complexes, which is in accordance with the previous experimental and theoretical studies (Freedberg et al., 1998; Zoete et al., 2002; Hou and Yu, 2007; Agniswamy et al., 2016). However, the flexibility of the G27 and A28 residues of the catalytic site (chain-A) was decreased in MUT-Pr-D. Moreover, compared with the WT-Pr-D, the MUT-Pr-D showed a significant decrease in the flexibility of residues in the fulcrum (Y14, I15, G16, G17, Q18, L19, and R20), flap-elbow (I36, N37, and L38) and cantilever (Q61, V62, and V63) regions of chain-A. However, the flexibility of the flap-tip of chain-A (residues V47, I50, G51, G52, F53, and M54) and flap-elbow (residues G40’ and Y41’) of chain-B of the MUT-Pr-D was remarkably increased compared with that of WT-Pr-D. It has been shown that increasing the flap-tip flexibility facilitates the opening of the active site gate, which consequently leads to the release of the inhibitor from the active site (Nicholson et al., 1995; Badaya and Sasidhar, 2020; Liu et al., 2020).

These 10 superimposed trajectories snapshots of WT-Pr-D and MUT-Pr-D with 2 ns intervals are shown in Figure 1C. As seen, the flap-elbow, fulcrum, cantilever, and flap-tip regions in chain-A and the flap-elbow region in chain-B experience the most conformational changes as a result of mutations. It is worth noting that the substituted residues in MUT-Pr-D were I13V and K20R (in the fulcrum), E35D, M36I, S37N, and R41K (in the flap-elbow), K43T, I47V, and I54M (in the flap-tip), and I62V, L63V, A71V, I72T, G73S, and T74P (in the cantilever region) residues (Supplementary Figure S1C).

### 3.3 Principal component analysis

In order to extract the essential dynamics, principal component analysis (PCA) was performed (David and Jacobs, 2014). Firstly, the rotational and translational movements of systems were removed by superimposing all of the structures on the reference structure. Then, the covariance matrix, eigenvector matrix, and eigenvalues matrix were constructed.

**FIGURE 2:**
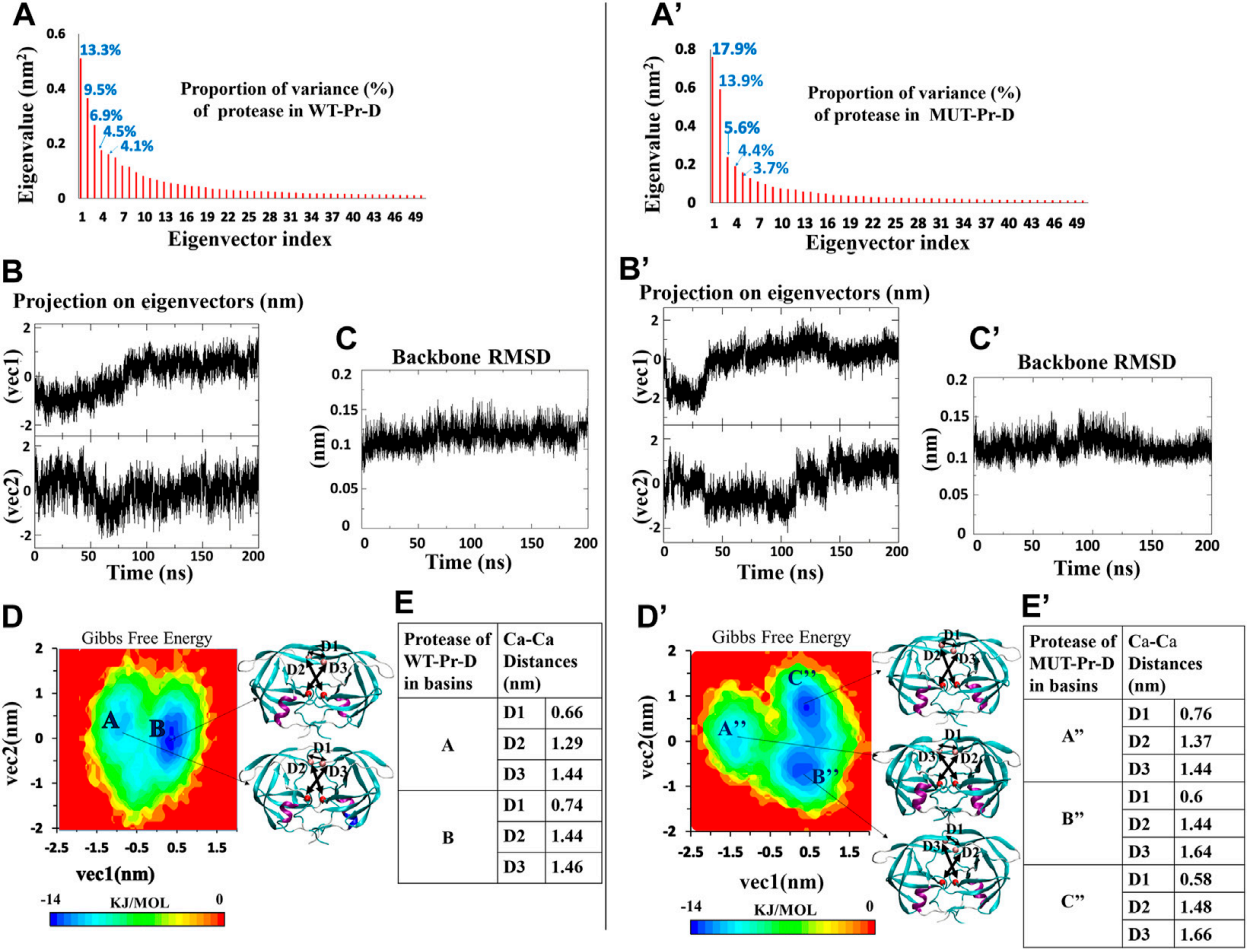
PCA for WT-Pr (left) and MUT-Pr (right) in complex with DRV, **(A,A’)** Eigenvalue for each corresponding eigenvectors (first 50 eigenvectors) and proportion of the variance with respect to corresponding eigenvectors; **(B,B’)** The projection of trajectory on the two first eigenvectors (PCs); **(C,C’)** The backbone RMSD for the proteases; **(D,D’)** The free energy landscape obtained from first two eigenvectors and protease structures having the lowest free energy; **(E,E’)** Distances between C_α_ of the residues I50-I50’: D1, D25-I50: D2 and D25′-D50’: D3 for the protease structures in the low-energy basins.

**FIGURE 3:**
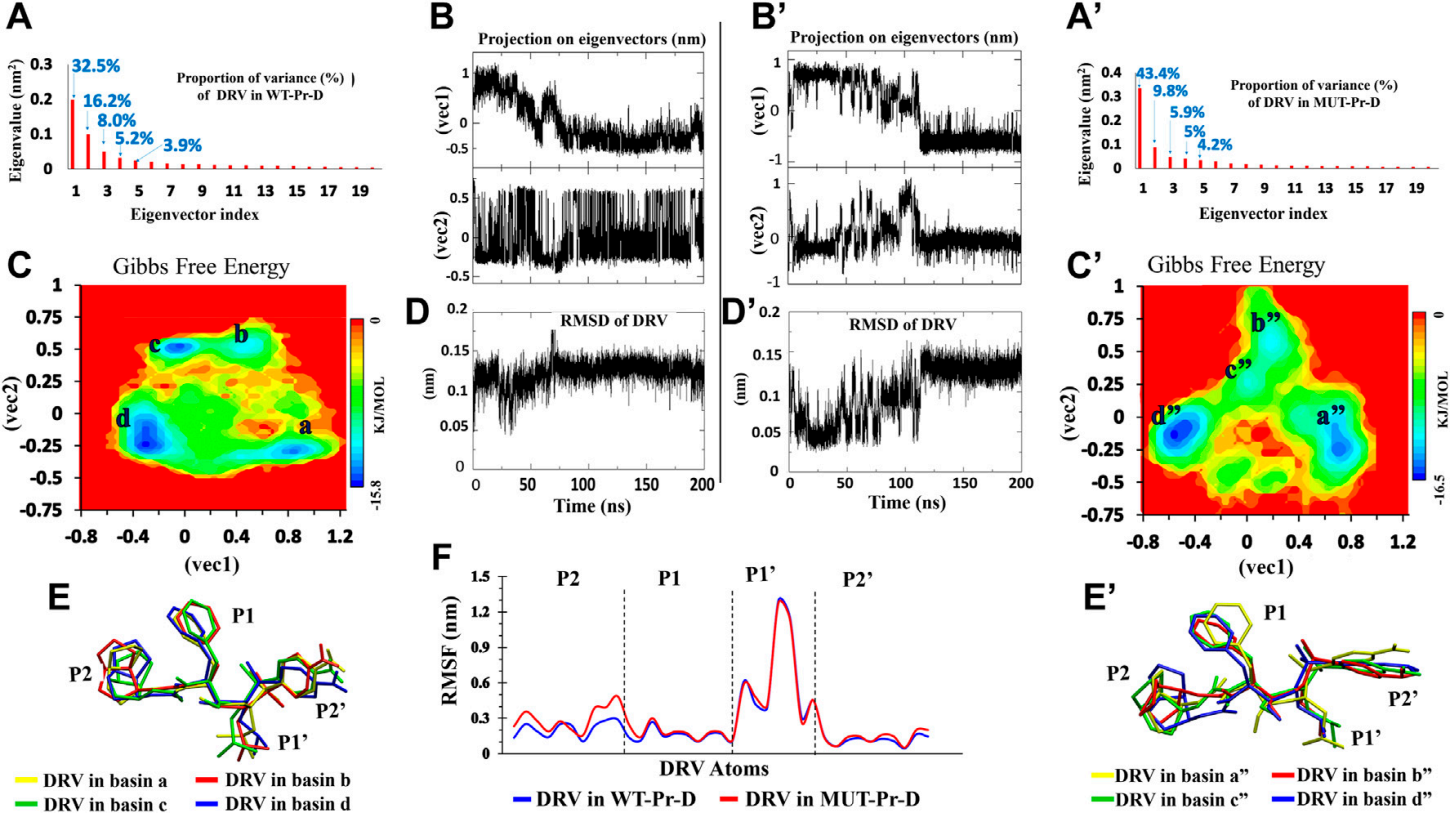
PCA for DRV in complex with WT-Pr-D (left) and MUT-Pr-D (right). **(A,A’)** The Eigenvalue for the first 20 eigenvectors and proportion of the variance with respect to corresponding eigenvectors; **(B,B’)** The projection of trajectory on the two first eigenvectors (PCs); **(C,C’)** The free energy landscape obtained from first two eigenvectors; **(D,D’)** The RMSD of DRV; **(E,E’)** The snapshot of lowest energy conformation of DRV in each basins; **(F)** The RMSF of non-hydrogen atoms of DVR’s moieties.

For the WT-Pr and MUT-Pr in complex with DRV, trajectories were projected separately on the first five eigenvectors (Supplementary Figure S3), and from these data, the first two eigenvectors of WT-Pr, MUT-Pr, and DRV showed a significant proportion of trajectory variance (Figures 2A,2A’, 3A,A’). So, it was rational to use them to extract the energy basins.

The two-dimensional Gibbs free energy landscape was calculated through Eq. 1, where P is the joint probability density function of the data along with the two principal components and T, K_B_, V1, and V2 are the absolute temperature (310 K), the Boltzmann constant, eigenvector 1 and eigenvector 2, respectively (Papaleo et al., 2009).

$$\Delta G_{\left(\mathrm{V1},\mathrm{V2}\right)}=-k_{B}T\ln P_{\left(\mathrm{V1},\mathrm{V2}\right)}$$ (1)

The free energy landscape of WT-Pr in a complex with DRV includes two low-energy basins (Figure 2D). At the beginning of the simulation, protease in WT-Pr-D was often found in local basin A (5–50 ns). During 50–80 ns, it transferred to the final basin B, and from 80 to 200 ns, it remained in basin B (Figures 2B and D). However, the free energy landscape of MUT-Pr in complex with DRV included three low-energy conformational basins (i.e., A”, B”, and C” Figure 2D’). So, the protease in MUT-Pr-D showed more conformational changes, which is consistent with a higher mean RMSD value (0.01 nm), as depicted in Figures 2C and C’ and mentioned in Section 3-1. At the beginning of the simulation (0–30 ns), the MUT-Pr was often found in local basin A” (high free energy relative to the other two basins). From 35 to 110 ns, it was found in basin B” and remained in this basin and then, during 110–140 ns, it transferred to final basin C” and until 200 ns remained in this basin (Figures 2B’and D’).

The C_α_ distances of D1 (I50–I50’), D2 (D25–I50), and D3 (D25’–I50’) were investigated to estimate the extent of the flap opening (Okimoto et al., 2001; Badaya and Sasidhar, 2020). These distances were analyzed for proteases inside each basin of WT-Pr-D (Figure 2E) and MUT-Pr-D (Figure 2E’). As seen in Figure 2E, for the WT-Pr-D, during the transition from basin A to B, distances of D1, D2, and D3 were increased by 0.08, 0.15, and 0.02 nm, respectively. On the other hand, for MUT-Pr-D (Figure 2E’), during the transition from basin A” to B”, the distance of D1 was decreased by 0.16 nm and distances of D2 and D3 were increased by 0.07 and 0.20 nm, respectively. Also, during the transition from basin B” to C”, the distance of D1 was decreased by 0.02 nm and the distances of D2 and D3 were increased by 0.04 and 0.02 nm, respectively (Figure 2E’). Finally, through comparing the WT-Pr-D and MUT-Pr-D at their final energy basins (B and C”, respectively), it was observed that D1 was decreased by 0.16 nm, which indicates that the flap-tips were opened. This opening occurred as a result of increasing the flexibility of the flap-tips as discussed in Section 3.2. Besides, the D2 and D3 distances were increased by 0.04 and 0.20 nm, respectively, ascribed to a slight increase in the active site space.

The DRV free energy landscape in complex with WT-Pr and MUT-Pr was also analyzed (Figures 3C and C’, respectively). As seen, four low-energy basins were observed for DRV in complex with WT-Pr, namely a, b, c, and d (Figure 3C). DRV in complex with MUT-Pr also showed four low-energy basins, namely a”, b”, c”, and d” in the trajectory space (Figure 3C’). In the WT-Pr-D, at the first 35 ns, although DRV was mainly found in the local basin a, it sometimes jumped to local basin b and came back again (Figure 3B). As a result of this transition, the structure was modified and RMSD value decreased by ∼0.1 nm (Figure 3D). After 35 ns, DRV was found dominantly in the local basin b and remained in it until 50 ns. From 50 to 55 ns, it overcame the energy barrier in basin b and was found in basin c. From 55 to 200 ns, DRV dominantly was found in basin d. However, sometimes jumped to basin c and came back again.

Figure 3C’ shows the DRV free energy landscape in complex with the MUT-Pr. In the first 80 ns, DRV was found dominantly in basin a” and sometimes jumped to basins b” and c”, then coming back to the primary structure. So, its structure was frequently changed, which was accompanied by ∼ 0.12 nm difference in the RMSD value (Figure 3D’). From 80 to 110 ns, the DRV structure was found in basins b” and c”. The difference in RMSD for this conformational transition was found to be ∼0.12 nm. After 110 ns, DRV was found in the basin d” and remained in this basin until 200 ns. Through this transition, its structure was modified and its RMSD increased by ∼0.1 nm.

In order to follow the conformational fluctuation of DRV in complex with WT-Pr and MUT-Pr, the RMSF of its heavy atoms (Figure 3F) was obtained and its structure in each of the basins is illustrated (the main chemical moieties of DRV, labeled as P1, P1’, P2, and P2’) (Figures 3E and E’). As seen in Figure 3F, although the P1’ moiety of DRV showed high flexibility in both WT-Pr-D and MUT-Pr-D, the other three moieties (P1, P2, and P2’) presented a low fluctuation, suggesting the presence of extensive interactions of these moieties with the active site residues of the protease. However, compared with WT-Pr-D, the P2 moiety of DRV in MUT-Pr-D showed more flexibility as a consequence of decreased interactions with the active site residues. Besides, according to Figures 3E and E’, during the transition from the first basin to the last one (a” to d”), the conformation of the P2 moiety of DRV in MUT-Pr-D experienced a significantly more change than that of the WT-Pr-D. This data is in good agreement with the RMSD results, in which DRV in MUT-Pr-D complex showed a more fluctuation in the RMSD value (Figures 3D and D’). The greater conformational change of DRV could be ascribed to the less compact active site cavity in MUT-Pr-D (as discussed earlier in this section), which provided more space for DRV motion.

**FIGURE 4:**
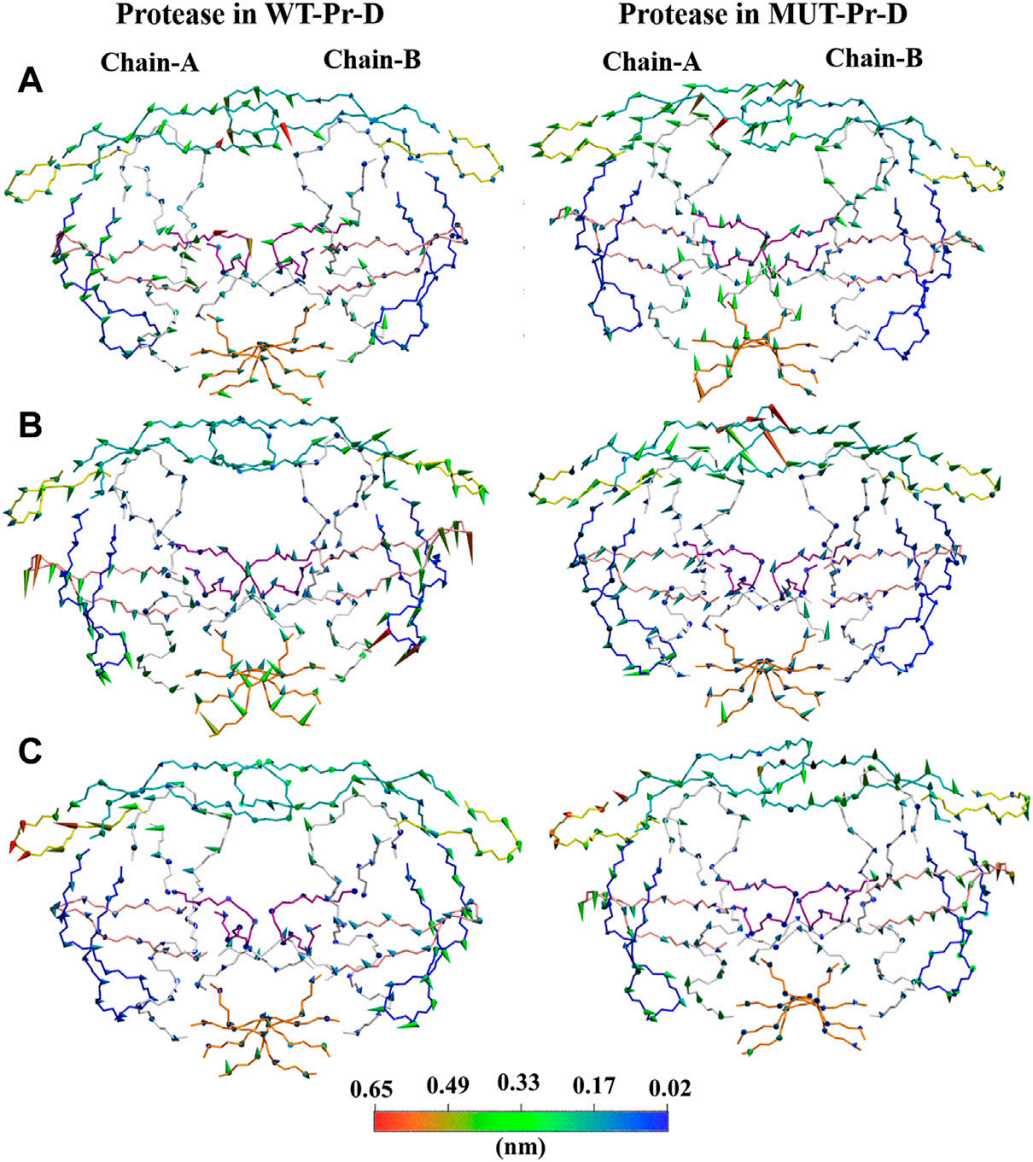
Projections of motions along first [**(A)**; PC1], second [**(B)**; PC2] and third [**(C)**; PC3] eigenvectors for C_α_ atoms of WT-Pr-D and MUT-Pr-D. DRV is not shown in the picture. The proteases segments are depicted in different colors; the active site by purple, the flap-elbows by yellow, the flap-tips by cyan, the fulcrums by pink, the cantilevers by blue, and the interfaces by orange colors. The direction of the cones describes the direction of motions and their lengths are correlated with the magnitude of motions, which was also indicated by the color gradient scale.

In order to extract the essential dynamics of proteases in WT-Pr-D and MUT-Pr-D, both of the trajectories were projected onto their first three eigenvectors (PC1, PC2, and PC3) and the main motions during 200 ns were obtained (Figure 4). PC1 involved a lot more structures in the trajectory as compared to PC2, and PC2 involved more structures than PC3. The PCA analysis showed that the effect of mutations on the motions of chain-A was greater than that of chain-B, which is in accordance with the RMSF data. As seen in Figure 4, the movements of the fulcrum, flap-elbow, and cantilever of chain-A in the MUT-Pr-D were significantly decreased relative to the WT-Pr-D, but the motions of flap-tip (chain-A) were increased. As seen in Figure 4A, the movements of fulcrum and cantilever (chain-A) in MUT-Pr-D were decreased relative to the WT-Pr-D, but the flap-elbow motion (chain-A) in MUT-Pr-D was increased. However, by considering PC2 and PC3 analysis, it is obvious that the total motion of the flap-elbow (chain-A) in MUT-Pr-D was decreased relative to that of the WT-Pr-D. These results are consistent with the RMSF data. Besides, the motion of cantilever, fulcrum (chain-A), and the flap-tips in WT-Pr-D were directed toward the protease active site. However, in MUT-Pr-D, these movements were directed outward from the active site. As seen in Figure 4B, the PC2 shows big downward motions of the flap-elbows, fulcrums, and cantilevers of WT-Pr-D (in the same direction) and a significant big motion of the flap-tips (chain-A) of MUT-Pr-D, which created a rotation (curling) in its conformation. The interface regions have a loop structure and inherently should be more flexible that is illustrated in PC1 and PC2 and is not very important in protease function. As seen in Figure 4C, the PC3 didn’t involve significant motions.

So, in the WT-Pr-D, the regions surrounding the active site moved in a way that led to a closed conformation. Besides, in the WT-Pr-D, the flap-tip residues move toward each other and to the active site (Figure 4A), while in MUT-Pr-D the flap-tip and flap-elbow of chain-A tend to move in a way that leads to an increase in the flap-tip opening (Figure 4A) and flap-tip of chain-A tend to rotate largely that change significant conformational changes (Figure 4B). These are signs which show that the MUT-Pr-D tends to form a semi-open conformation (Perryman et al., 2004; Perryman et al., 2006; Kunze et al., 2014; Ung et al., 2014; Liu et al., 2020).

### 3.4 Distance and radial distribution function analysis

**FIGURE 5:**
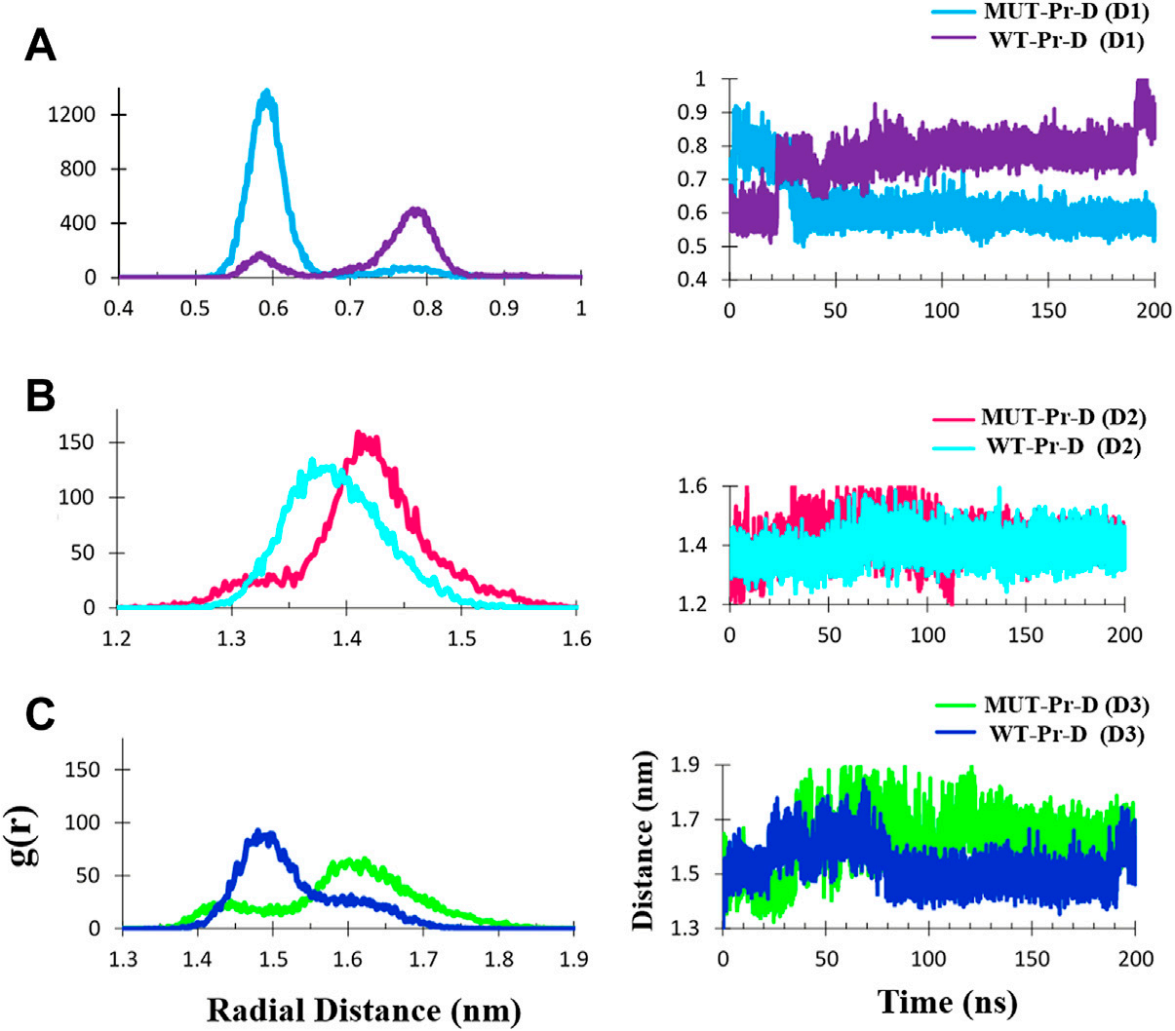
The C_α_- C_α_ radial distribution functions (RDF) and distances during 200 ns MD simulation for MUT-Pr-D and WT-Pr-D were measured for D1 **(A)**, D2 **(B)**, and D3 **(C)**.

In addition to obtaining the D1, D2, and D3 distances from the lowest free energy structures in the energy basins, these distances were also determined during the total simulation time. For this purpose, distances of D1, D2, and D3 in both the WT-Pr-D and MUT-Pr-D complexes were obtained from the radial distribution function (RDF) (Figure 5).

The average ± SD distance of D1 was ∼0.62 ± 0.07 and 0.76 ± 0.07 nm in the MUT-Pr-D and WT-Pr-D, respectively. The most probable distances were ∼0.58–0.65 nm and 0.75–0.8 nm for MUT-Pr-D and WT-Pr-D, respectively (Figure 5A). As seen, the flap-tip to flap-tip average distance was about 0.14 nm smaller in MUT-Pr-D. Considering that residues I50 and I50’ were located in the cap of the flap-tips, when the flap-tips got far away from each other along the longitudinal axis, these residues became closer to each other (Supplementary Figure S4). Based on the previous reports, the flap-tips separation of more than 1 nm was considered an open conformation and from 0.6 to 1 nm a semi-open conformation (Badaya and Sasidhar, 2020; Liu et al., 2020; Sk et al., 2020). However, in our 200 ns simulation, the flap-tips conversion from closed to semi-open conformation has not been shown directly because it acquires microsecond to millisecond time to occur as NMR experiments have revealed (Ishima et al., 1999; Freedberg et al., 2002; Katoh et al., 2003).

The D2 distance was measured during the 200 ns MD simulations. The mean ± SD distances of D2 were ∼1.42 ± 0.05 and 1.39 ± 0.04 nm for MUT-Pr-D and WT-Pr-D, respectively. The most probable distances (RDF) were ∼1.40–1.45 nm and 1.35–1.40 nm for MUT-Pr-D and WT-Pr-D, respectively (Figure 5B). So, in chain-A of the MUT-Pr-D, the average distance between the flap-tip and active site was increased by ∼0.03 nm as a result of mutation. This increase was even greater in chain-B. The mean ± SD distance values of D3 were ∼1.60 ± 0.09 and 1.50 ± 0.07 nm for MUT-Pr-D and WT-Pr-D, respectively. The most probable distances were ∼1.58–1.65 nm and 1.45–1.55 nm for MUT-Pr-D and WT-Pr-D, respectively (Figure 5C). Thus, the average flap-tip to active site distance in chain-B of the MUT-Pr-D has increased on average by ∼0.10 nm. As seen, the opening movements of the flap-tips were observed as a result of mutations leading to the conversion of the closed conformation to a semi-open conformation and releasing inhibitors from the active site region (Ding et al., 2008; Badaya and Sasidhar, 2020).

### 3.5 The hydrophobic interactions

In ligand-bonded WT-Pr, the hydrophobic interactions between I50 and its adjacent residues I47’/I54′ as well as hydrophobic interactions between I50’ and its adjacent residues I47/I54 (Supplementary Figure S5) play an important role in the retention of the flap-tips in a closed conformation, which consequently traps DRV in the active site (Lange and Grubmüller, 2006; Yu et al., 2015; Pawar et al., 2019). Previous experimental analysis of the extensive statistical community that suffers from AIDS disease has revealed that I47V, I54M, I47’V, and I54’M substitutions are the most prevalent substitutions in the resistant strains of DRV (Wheeler et al., 2010). So, the interactions between these residues were compared in MUT-Pr-D and WT-Pr-D during 200 ns MD simulations. For this purpose, RDF and the distance between C_α_ atoms of I50’ and I47/I54 (Supplementary Figures S5 and S6) and between I50 and I47’/I54’ in both complexes were calculated, in which I47 was substituted by V and I54 was substituted by M in chain-A and B of MUT-Pr-D (Supplementary Figures S5 and S7).

The average distance ± SD between I50’ and V47 in MUT-Pr-D and between I50’ and I47 in WT-Pr-D were calculated to be ∼0.92 ± 0.08 nm and 0.88 ± 0.05 nm, respectively (Supplementary Figure S6A). As seen, in MUT-Pr-D, the average distance was increased by ∼0.04 nm compared to that of WT-Pr-D. The most probable distances were calculated to be ∼0.81–1.00 nm and 0.83–0.92 nm for MUT-Pr-D and WT-Pr-D, respectively (Supplementary Figure S6A). The average distance ± SD between I50’ and M54 in MUT-Pr-D and between I50’ and I54 in WT-Pr-D were calculated to be ∼0.7 ± 0.05 and 0.75 ± 0.06 nm, respectively (Supplementary Figure S6B). So, in MUT-Pr-D, the average distance decreased by about 0.05 nm relative to that of WT-Pr-D. The most probable distances were calculated as ∼0.65–0.72 Å and 0.73–0.78 Å for MUT-Pr-D and WT-Pr-D, respectively (Supplementary Figure S6B).

The average distance ± SD between I50 and V47’ in MUT-Pr-D and between I50 and I47’ in WT-Pr-D was calculated to be ∼0.92 ± 0.09 and 0.81 ± 0.05 nm, respectively, showing that the average distance increased about 0.11 nm relative to that of WT-Pr-D due to mutations (Supplementary Figure S7A). The most probable distances were calculated to be ∼0.83–0.87, 0.98–1.03, and ∼0.78–0.83 nm for MUT-Pr-D and WT-Pr-D, respectively (Supplementary Figure S7A). The average distance ± SD between I50 and M54’ in MUT-Pr-D and between I50 and Ile54’ in WT-Pr-D was calculated to be 0.82 ± 0.07 and 0.75 ± 0.03 nm, respectively (Supplementary Figure S7B). So, this distance increased ∼0.07 nm relative to that of WT-Pr-D due to mutations. The most probable distances were calculated to be ∼0.80–0.85 and 0.73–0.77 nm for MUT-Pr-D and WT-Pr-D, respectively (Supplementary Figure S7B). However, the distance between I50’ and M54 in MUT-Pr-D was decreased (Supplementary Figure S6B), showing increased hydrophobic interaction. However, three other mentioned hydrophobic interactions between the flap-tips were decreased in MUT-Pr-D. So, the flap-tip separation relative to each other occurred due to the reduction of hydrophobic interactions (Yu et al., 2015). As a result, the active site gateway could be opened easier in MUT-Pr-D than in WT-Pr-D (Badaya and Sasidhar, 2020; Liu et al., 2020).

### 3.6 Comparing the darunavir–proteases interactions

In order to compare the binding potency of DRV to WT-Pr and MUT-Pr, the binding energies in the last basins B and C” were calculated by MM-PBSA approach (Genheden and Ryde, 2015) (Table 1). In MUT-Pr-D, compared with WT-Pr-D, the part of the binding energy related to the electrostatic interactions (ΔE_electrostatic_) was unfavorable by 13.6 kj mol^−1^ averagely. The van der Waals (VDW) energy contribution was also unfavorable by ∼25.0 kj mol^−1^ averagely (Table 1; Supplementary Figure S8). The polar solvation energy (∆G_Polar-solvation_) contribution of the binding energy was favorable by 21.5 kj mol^−1^ and the non-polar solvation energy contribution (∆G_SASA_) was almost the same in both systems (Table 1). So, it can be concluded that in MUT-Pr-D, the electrostatic and VDW interactions between the protease and DRV were decreased. However, the polar solvation energy partly compensates for this reduction. Totally, the enthalpy of the protease-DVR interaction in WT-Pr-D was ∼17.3 kj mol^−1^ more favorable than that of MUT-Pr-D.

**TABLE 1:** Comparison of binding energy components between the protease and DRV in the WT-Pr-D and MUT-Pr-D systems. Also, other experimental and simulation studies about DRV binding to some mutant HIV-1 proteases were shown.

Energy components	WT-Pr-D	MUT-Pr-D
∆E_vdw_	−289.6 ± 15.3	−264.56 ± 18
∆E_elec_	−88.7 ± 10.7	−75.1 ± 13.1
∆G_polar-solv_	249.4 ± 12.6	227.9 ± 16
∆G_SASA_	−26.4 ± 0.7	−26.2 ± 0.9
∆H	−155.3 ± 16.5	−138 ± 18.2
∆H^exp^	−68.33 ± 0.12^a^−50.41^b^	−31.25 ± 0.04^a^
∆H^simulation^	−170.8 ± 16.37^c^	
−T∆S	10.6 ± 0.35	4.3 ± 0.22
−T∆S^exp^	2.5 ± 0.41^a^−12.9^b^	−20.83 ± 0.41^a^
−T∆S^simulation^	110.1 ± 31.3^c^	
∆G	−144.7	−133.7
∆G^exp^	−63.33^b^−55^d^−65.8 ± 0.41^a^	−52 ± 0.41^a^
∆G^simulation^	−61.12 35.3^c^	

All the energies are in KJ/mol. Total binding energies (∆G_MMPBSA:_ Molecular mechanics/Poisson-Boltzmann Surface Area) obtained from the sum of the ∆E_vdw_ (VDW interaction energy), ∆E_electrostatic_ (Electrostatic interaction energy in the gas phase), ∆G_polar-solvation_ (Polar solvation energy) and ∆G_SASA_ (Non-Polar solvation energy) energies; -TΔS, total entropy contribution; enthalpy, ΔH = ΔE_elec_ + ΔE_vdw_ + ΔG_polar-solv_+ ΔG_SASA_; Gibbs free energy, ΔG = ΔH−TΔS.

a (Kožíšek et al., 2014).

b (King et al., 2004).

c (Meher and Wang, 2015).

d (Kovalevsky et al., 2006).

The quasi-harmonic (QH) approach (Brooks et al., 1995) was used to calculate the configurational entropies of the binding of DRV to WT-Pr and MUT-Pr. In this method, the atomic fluctuation matrix could be obtained from the snapshots of the trajectories based on the PCA analysis of the mass-weighted variance-covariance matrix (Hikiri et al., 2016). Table 1 shows the configurational entropies of the binding of DRV for both systems, obtained from PCA analysis on the two first eigenvectors. As seen, the entropy contribution (-T∆S) of the MUT-Pr-D decreased by ∼6.3 kj mol^−1^ compared with that of WT-Pr-D, which partly compensates for the unfavorable enthalpy of binding. By considering both the enthalpy and entropy contributions, the binding Gibbs free energy of binding (ΔG) of DRV to protease in WT-Pr-D was ∼10.8 kj mol^−1^ more favorable than that of MUT-Pr-D (Table 1; Supplementary Figure S8), due to the decrease of the VDW and electrostatic interactions in MUT-Pr-D as was denoted experimentally (Kožíšek et al., 2014) (Table 1). This is also consistent with another work based on the MD simulation, which showed that decreased WDV interactions are the main reason for the resistance of a mutant HIV-1 protease to DRV (Chen et al., 2015). So, the unfavorable enthalpy of binding of the DRV to MUT-Pr relative to WT-Pr leads to its resistance to DRV.

**FIGURE 6:**
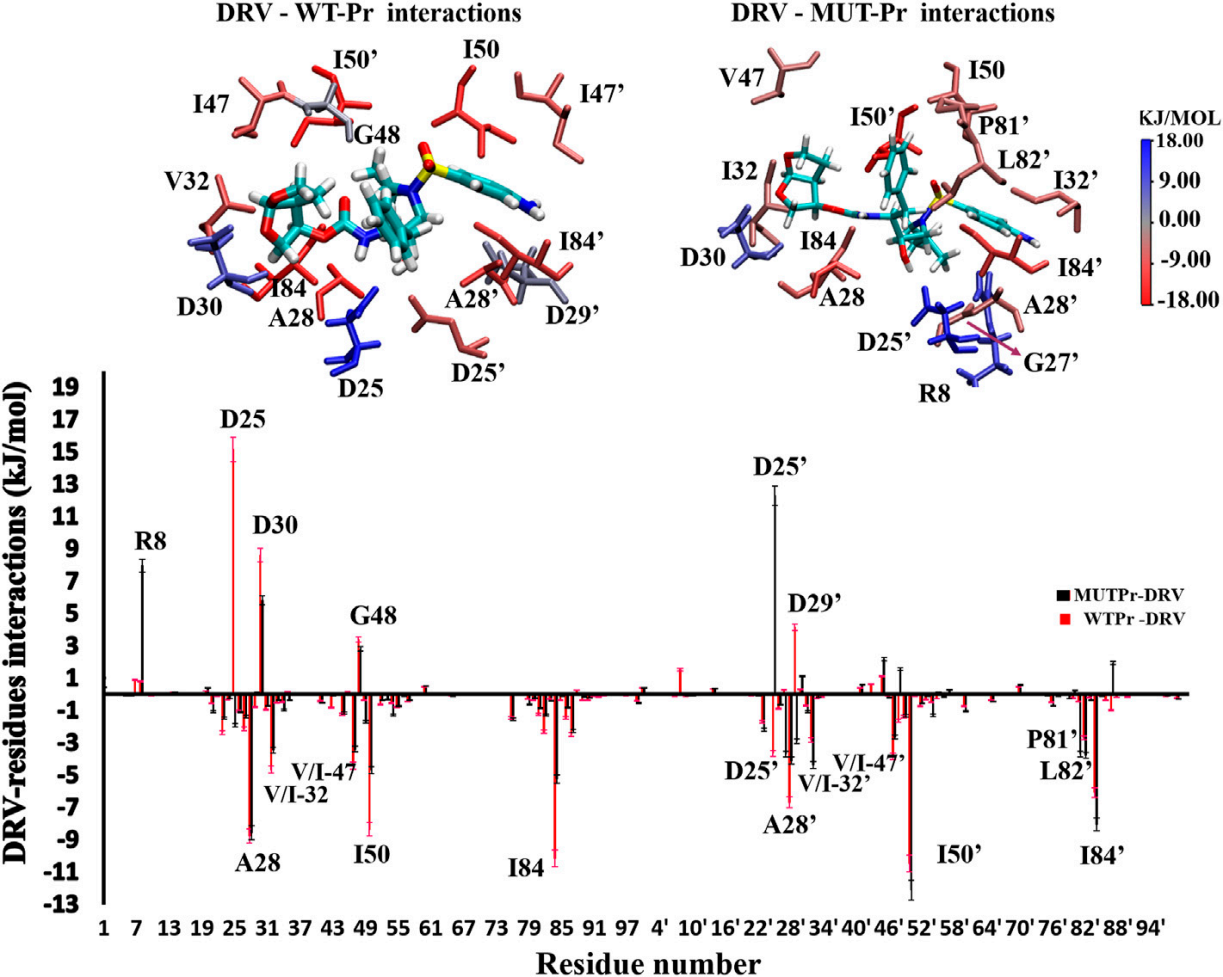
Down: residual spectrum of binding energy with DRV. Up: The group of interactions consists of residues in total with the binding energy equal to or more than +/−3 kj.mol^−1^ was be showed, and their binding potency was determined visually by color indicator.

In order to identify the residues that play an important role in the binding of the DRV to proteases, in WT-Pr-D and MUT-Pr-D the MM-PBSA approach was used (Supplementary Table S1; Figure 6). As seen, the residues A28, I50, I84, A28’, I50’, and I84’ of WT-Pr play an important role in binding to the DRV, which is consistent with the previously reported results (Meher and Wang, 2012; Chen et al., 2018). On the other hand, the energy analysis also showed the main unfavorable binding energy of D25, D30, D29’, and G48 residues to DRV in WT-Pr. Besides, as seen in Figure 6, the enthalpy-unfavorable binding of DRV to MUT-Pr relative to WT-Pr is mainly related to the R8, I50, I84, D25’, and A28’ residues, which induced a remarkable increase in the enthalpy value (Supplementary Table S1; Figure 6). In order to determine the residues that mainly affect the weak binding of DRV to MUT-Pr, the binding energy of these residues was decomposed (Supplementary Table S1). As seen, the main forces that lowered the binding energy to DRV in I50, I84, and A28’ were VDW and electrostatic interactions, and the contribution of polar solvation and nonpolar solvation interactions was far less important. For R8, in addition to the VDW and electrostatic interactions, the polar solvation interactions also play an important role. For D25’, only the polar solvation interactions contribute unfavorably to the binding energy. The binding energy difference between D25 and D25’ with DRV in WT-Pr-D was large (Figure 6). The favorable binding energy of D25’ with DRV in WT-Pr-D could be attributed to the favorable polar solvation energy of ∼−2 kj mol^−1^ and electrostatic and VDW binding energy of ∼−1.5 kj mol^−1^ (Supplementary Table S1). The unfavorable binding energy of D25 with DRV in WT-Pr-D could be ascribed to the unfavorable polar solvation energy of ∼42 kj mol^−1^ (data not shown) despite the presence of favorable electrostatic and VDW binding energy of ∼−27 kj mol^−1^ (data not shown).

### 3.7 Hydrogen bonds between darunavir and HIV-1 proteases

**FIGURE 7:**
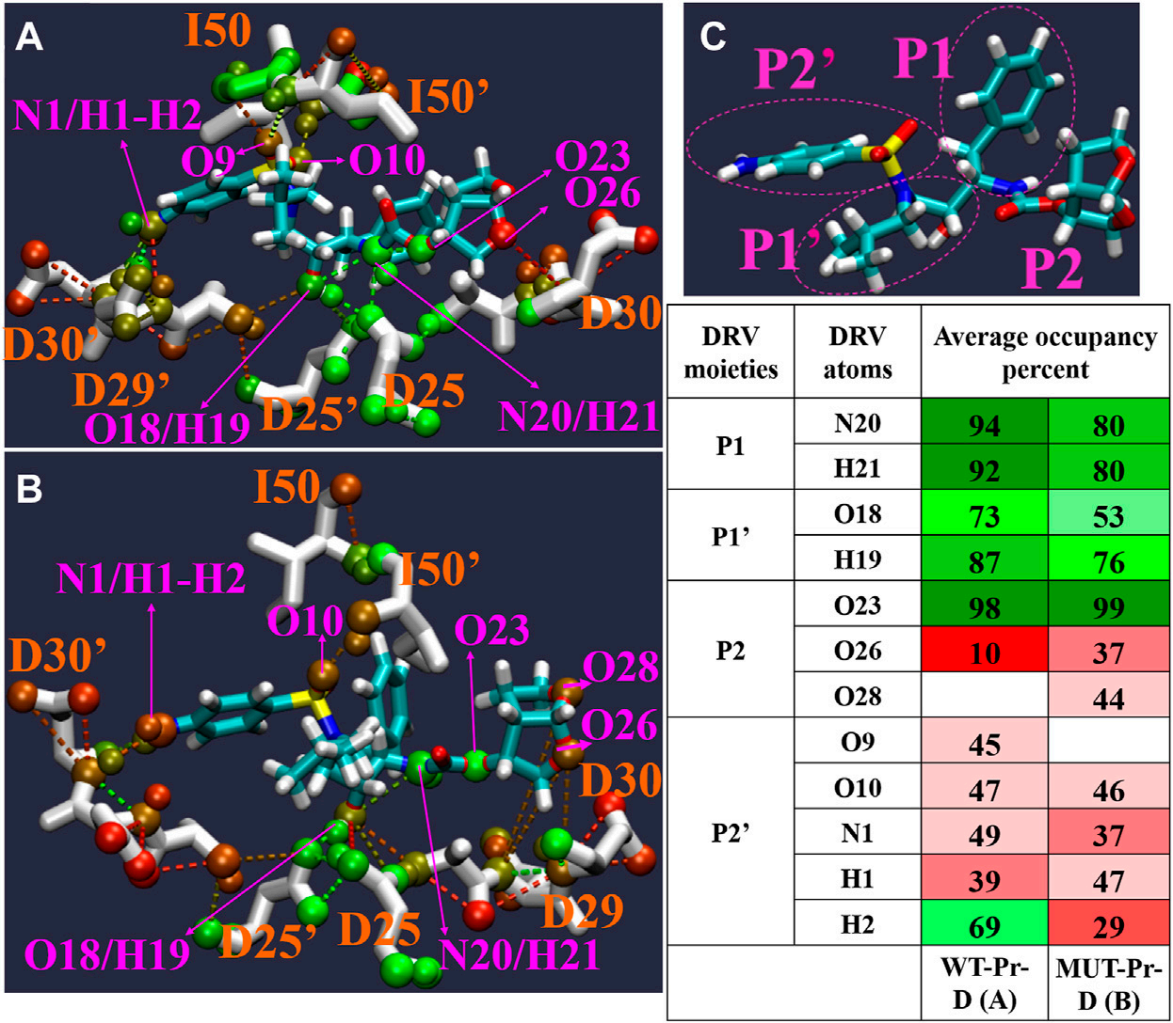
The Pattern of hydrogen bonds networks (HB) between DRV with WT-Pr **(A)** and MUT-Pr **(B)** in minimum energy basins B and C were indicated. **(C)** the main chemical moieties of DRV, labeled as P1, P1’, P2, and P2’. HBs percent in both basins are illustrated in table; red colors are for hydrogen bonds that their occupancy is lower than 50 percent, and green colors are for hydrogen bonds that their occupancy are higher than 50 percent. Only, those HBs that were formed at least in 10 percent of structures and angle between three atoms involving in HBs were between 0–60 degree were considered.

Hydrogen bond (HB) patterns between DRV and HIV-1 PR are important and can affect enzyme inhibition (Sayer et al., 2008; Huang et al., 2012; Huang et al., 2014). In order to investigate the effect of mutation on these interactions, HBs between DRV and HIV-1 PR from global minimum basins B and C” were analyzed by the Hbonanza program (Durrant and McCammon, 2011) program. Figure 7 shows the HBs data, shown by color gradient, in which red colors represent the HBs with an occupancy of less than 50 percent and green colors show the HBs with an occupancy of higher than 50 percent. In our analysis, only HBs in which the distance between hydrogen donor and acceptor atoms was equal or less than 0.35 nm and the angle of donor-H-acceptor atoms was between 0-60° have been considered. As seen in Figure 7, while N20 and H21 atoms of DRV (P1) formed HBs with D25 of WT-Pr by HB occupancy of more than 90%, they didn’t form any HBs with the MUT-Pr. The reported HBs for these atoms in MUT-Pr correspond to intramolecular HBs in DRV, as depicted in Figure 7B. The O18 and H19 atoms of P1’ site of DRV formed HBs with D25 and D25’ of both proteases. As seen in Figure 7, the HBs frequency in MUT-Pr decreased by 20% and 11% for O18 and H19 atoms, respectively. The O26 atom of DRV (P2 site) formed HBs with D30 of WT-Pr (∼10%) and D30 of MUT-Pr (∼37%). The O28 atom of DRV (P2 site) formed HBs only with D29 of MUT-Pr (∼44%). Neither WT-Pr nor MUT-Pr formed HBs with the O23 atom of DRV and only showed intramolecular HBs interactions. The O9 and O10 atoms (P2’ site) of DRV make HBs with the I50’ and I50 of WT-Pr, respectively. While in MUT-Pr, O10 maintained nearly identical HBs frequencies and O9 didn’t take part in the hydrogen bonding interaction with I50’ of DRV. The N1, H1 atoms (P2’) of DRV make HBs with both D29’ and D30’ of WT-Pr and D30’ of MUT-Pr, showing almost identical HBs occupancy. The H2 atom of DRV (P2’) formed HBs with D30’ of WT-Pr and MUT-Pr. However, this atom experienced ∼40 % decrease in the HB frequencies with the D30’ of MUT-Pr. So, in the MUT-Pr, the H2 atom of the NH_2_ group (P2’) accounts for much of the decrease in HBs. Briefly, our results showed that the hydrogen bonding network was disturbed in MUT-Pr, leading to a decreased potency of DRV binding to the active site. This could cause resistance against DRV (Chen et al., 2018; Henes et al., 2019), as discussed in Sections 3.3 and 3.4.

### 3.8 Radius of gyration

In order to assess the compactness of the protease active site cavity, the radius of gyration (g(r)) of the backbone atoms was calculated for residues 23–32, 46–54, and 78–87 in both chains (Supplementary Figure S9). As seen, the average ± SD g(r) values during the first 110 ns simulation time for MUT-Pr-D and WT-Pr-D were 1.05 ± 0.01 nm and 1.06 ± 0.01 nm, respectively. So, the average g(r) in WT-Pr-D was ∼0.01 nm larger than that of MUT-Pr-D. So, during the first 110 ns, the active site of MUT-Pr-D was more compact than that of WT-Pr-D. This shows that, during the first 110 ns, the active site of MUT-Pr-D was more compact than that of WT-Pr-D. However, after 110 ns, (in basins B and C”), the average g(r) and standard deviation for MUT-Pr-D and WT-Pr-D were calculated as 1.08 ± 0.007 nm and 1.05 ± 0.006 nm, respectively. Thus, the g(r) of the MUT-Pr-D active site increased slightly to 0.03 nm and became less compact than that of WT-Pr-Dr. So, at basin C” relative to basin B, the active site cavity space was increased. These results are in agreement with the other discussed results (in Sections 3.3 and 3.4).

## 4 Conclusion

In this study, the mechanism of resistance of a MUT-Pr strain against DRV was investigated. The PCA and RMSF analyses showed that in MUT-Pr-D, the flap-tips get away from each other, curl significantly, and become more flexible in chain-A. Based on the distance and RDF analysis, it was also shown that the average distance of the flap-tips from each other and from the active site increased in MUT-Pr-D, leading to conversion from a closed to a semi-open conformation. The increase in flap-tip (chain-A) to flap-tip (chain-B) distance occurred as a result of decreasing hydrophobic interactions in MUT-Pr-D. The PCA and g(r) analysis showed that the volume of the active site in MUT-Pr-D becomes a little larger, which was accompanied by a wide conformational change of DRV. This is because, in WT-Pr-D, the fulcrum, cantilever, and flap-tips move toward the active site core, while in MUT-Pr-D, these movements were slightly outward from the core of the active site. The binding energy analysis showed that the MUT-Pr resistance against the DRV is created by unfavorable binding enthalpy in the MUT-Pr-D relative to the WT-Pr-D, which overcomes the more favorable binding entropy in MUT-Pr-D. The MM-PBSA approach showed that the R8, I50, I84, D25’, and A28’ residues play a major role in increasing the enthalpy of binding *via* reducing van der Waals (VDW) and electrostatic interactions of the residues I50, I84, A28’, and van der Waals (VDW), electrostatic and polar solvation interactions of the residue R8 and polar solvation interaction of the residue D25’. As all of these residues are conserved in our mutant strain, the mutations indirectly drive resistance to DRV and are distal mutations. The HBs analysis indicated that mutations disturbed the hydrogen bonding network. Briefly, it can be concluded that resistance of MUT-Pr to DRV occurred due to changes in the structure and dynamics of the protease, which decreased the binding potency to the DRV.

## Data Availability

The raw data supporting the conclusions of this article will be made available by the authors, without undue reservation.

## Footnotes

1 FDA: Food and Drug Administration.
