# Supplementary material for: The structural, dynamic, and thermodynamic basis of darunavir resistance of a heavily mutated HIV-1 protease using molecular dynamics simulation
Source: Front Mol Biosci. 2022 Aug 15;9:927373. doi: 10.3389/fmolb.2022.927373 (PMC9420863; doi:10.3389/fmolb.2022.927373)
Supplement: Supplementary file 1 [file DataSheet1.docx]

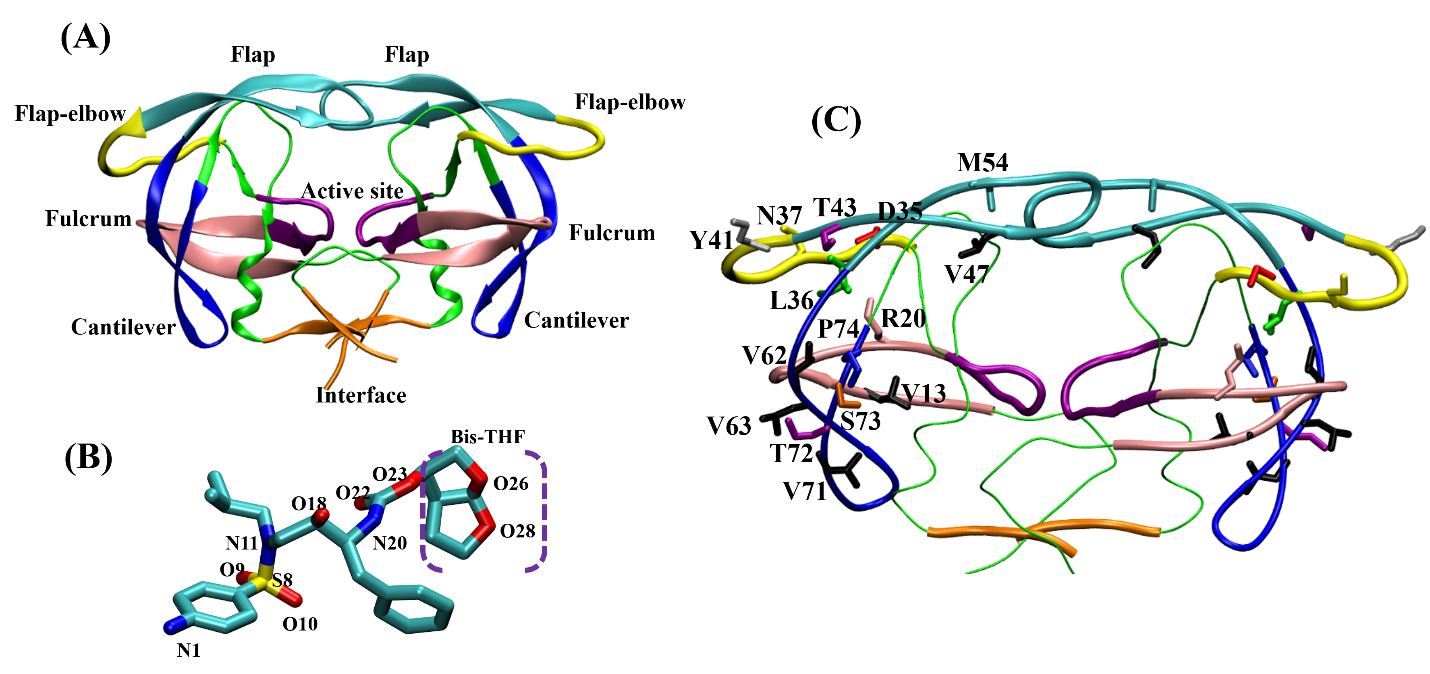


Fig. S1) (A): The six structural segments of the HIV-1 PR homodimer. Different segments have been identified by colors; (B): The labeled Darunavir atoms. The bis-tetrahydrofuran (THF) group is illustrated in a bracket; (C): The substituted residues in MUT-Pr-D. In both chain A and B, more than 90 percent of the substituted residues were placed in the fulcrum, flap-elbow, flap-tip and cantilever regions.


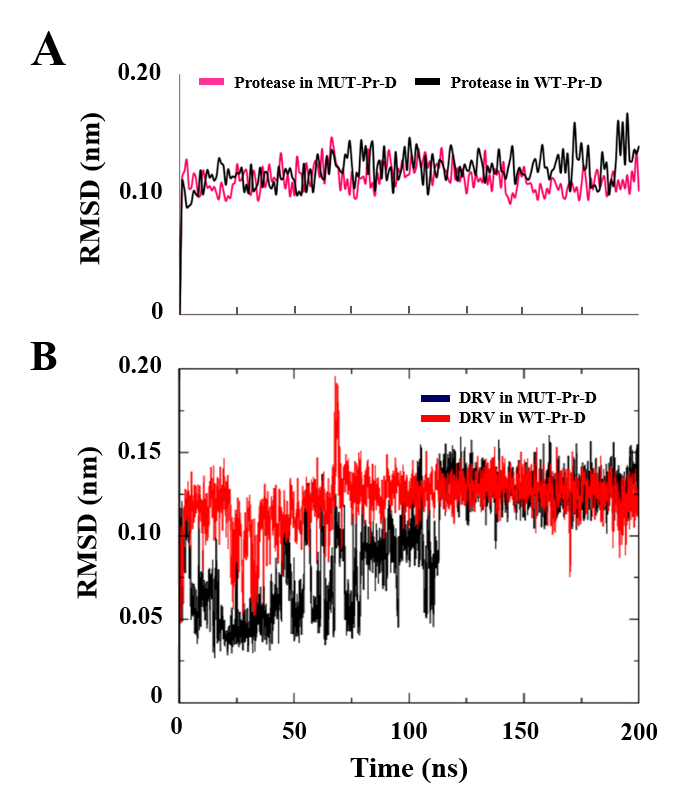


Fig. S2) (A) Root Mean Square Deviation (RMSD: nm) for MUT-Pr-D and WT-Pr-D backbone atoms during 200 ns by 1 ns interval; (B) RMSD of the heavy atoms of DRV in complex with MUT-Pr (black) and WT-Pr (red) during 200 ns.


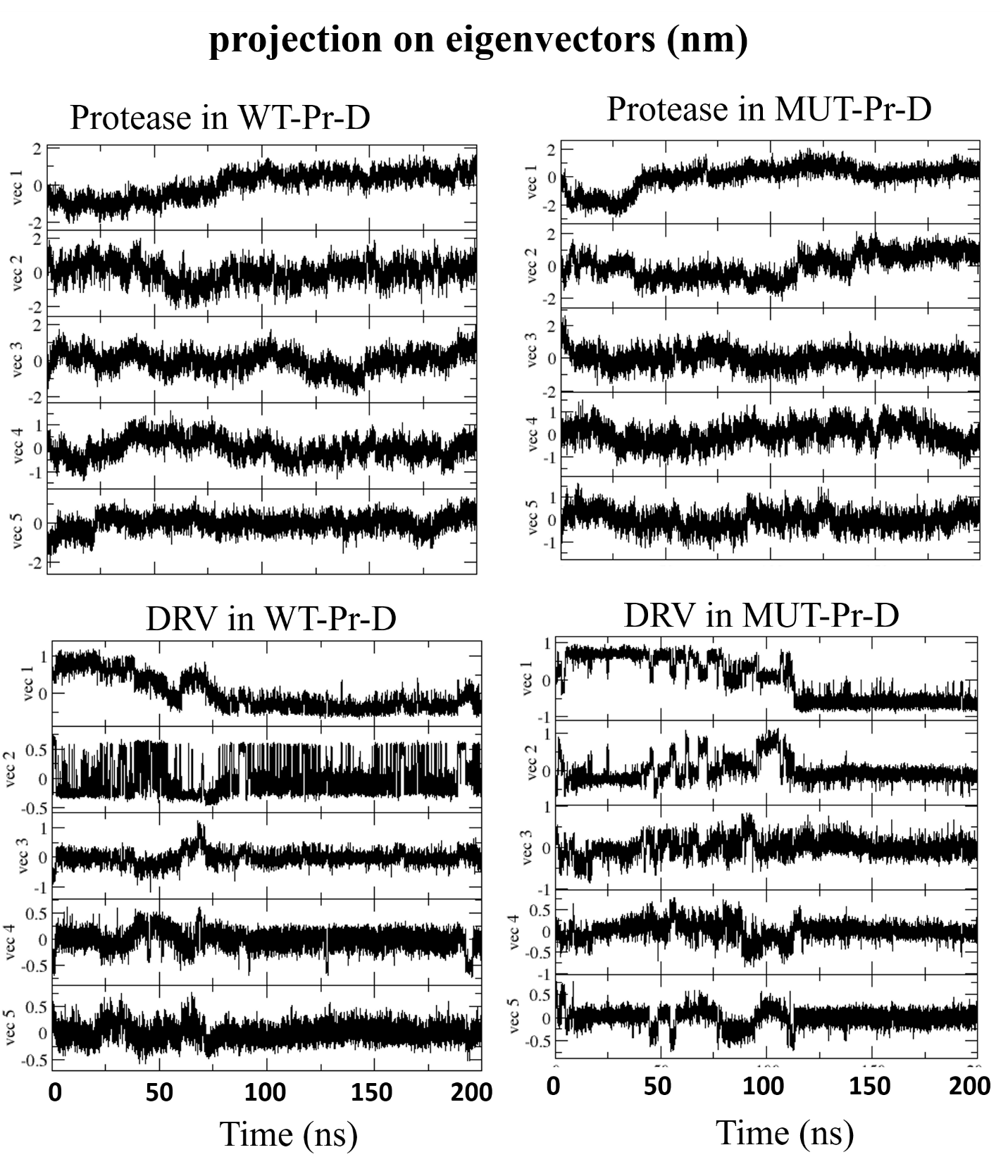


Fig. S3) The projection of trajectories on the first five eigenvectors for WT-Pr, MUT-Pr and the DRV in WT-Pr-D and MUT-Pr-D complexes.


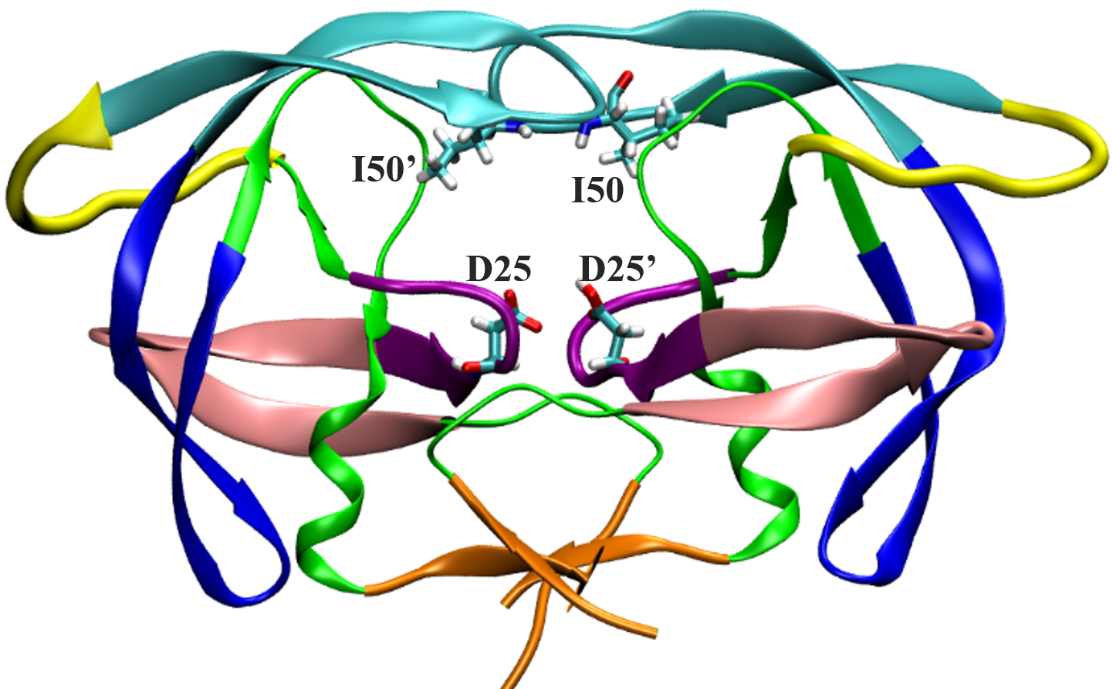


Fig. S4) Structure of WT-Pr-D. Only side chains of D25 and I50 in chains-A and B were shown. The distance between Ca atoms of these residues can indicate the size of active site cavity.


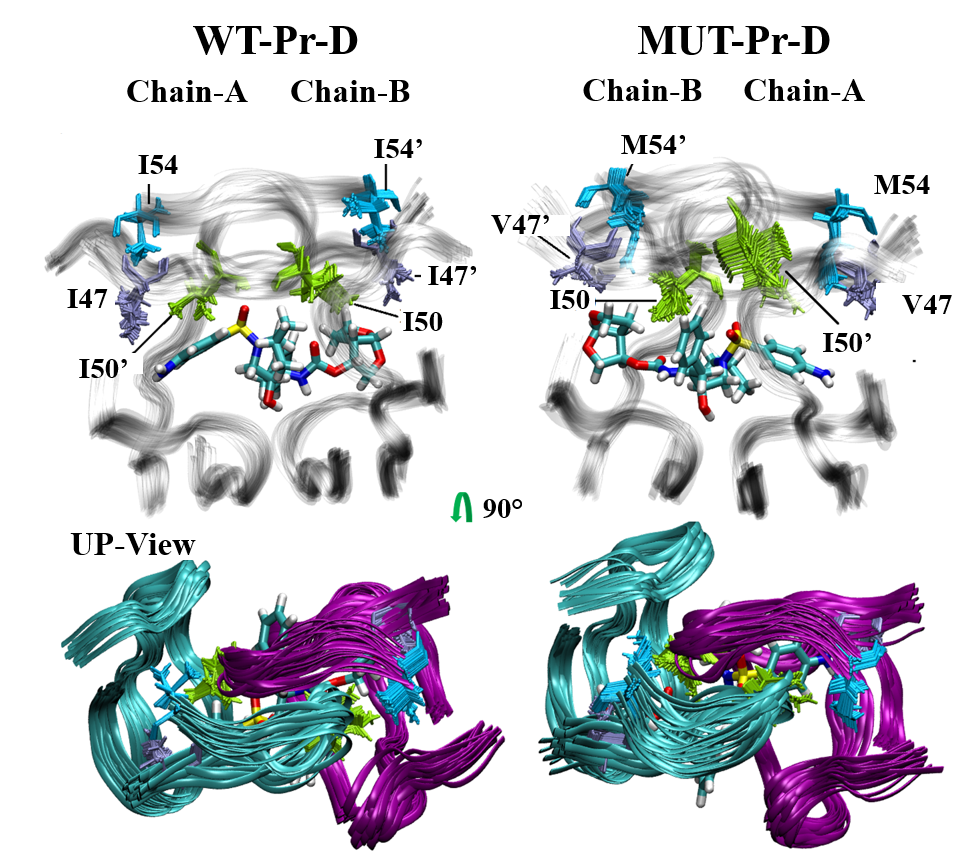


Fig. S5) The 20 superposed Snapshots of backbone of flap-tips and residues that are around DRV in WT-Pr-D (A) and MUT-Pr-D (B) with 10 ns intervals have been shown, hydrophobic residues in flap-tips are labeled in picture.


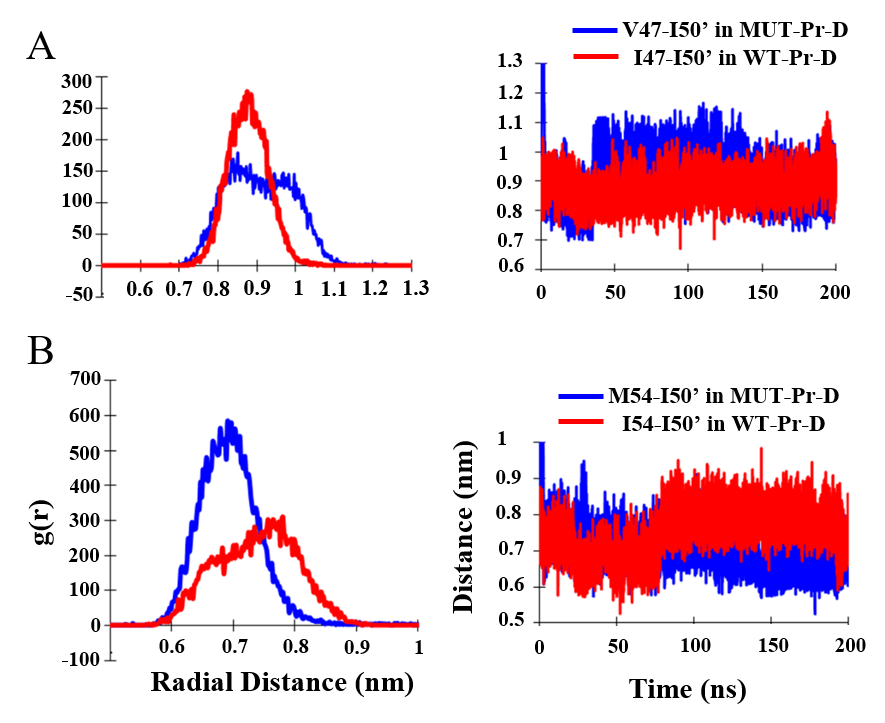


Fig. S6) Left, C_α_ radial distribution function; and Right, distance (nanometer) between flap-tip’s hydrophobic residues in MUT-Pr-D (blue) and WT-Pr-D (red).


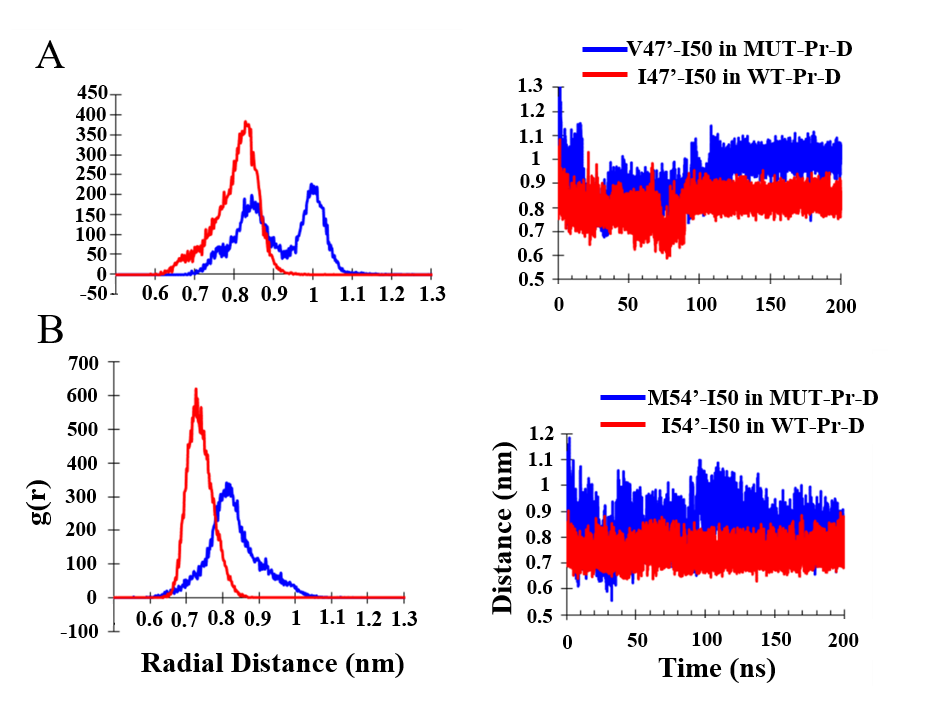


Fig. S7) Left, C_α_ radial distribution function; and Right, distance (nanometer) between flap tip’s hydrophobic residues in MUT-Pr-D (blue) and WT-Pr-D (red).


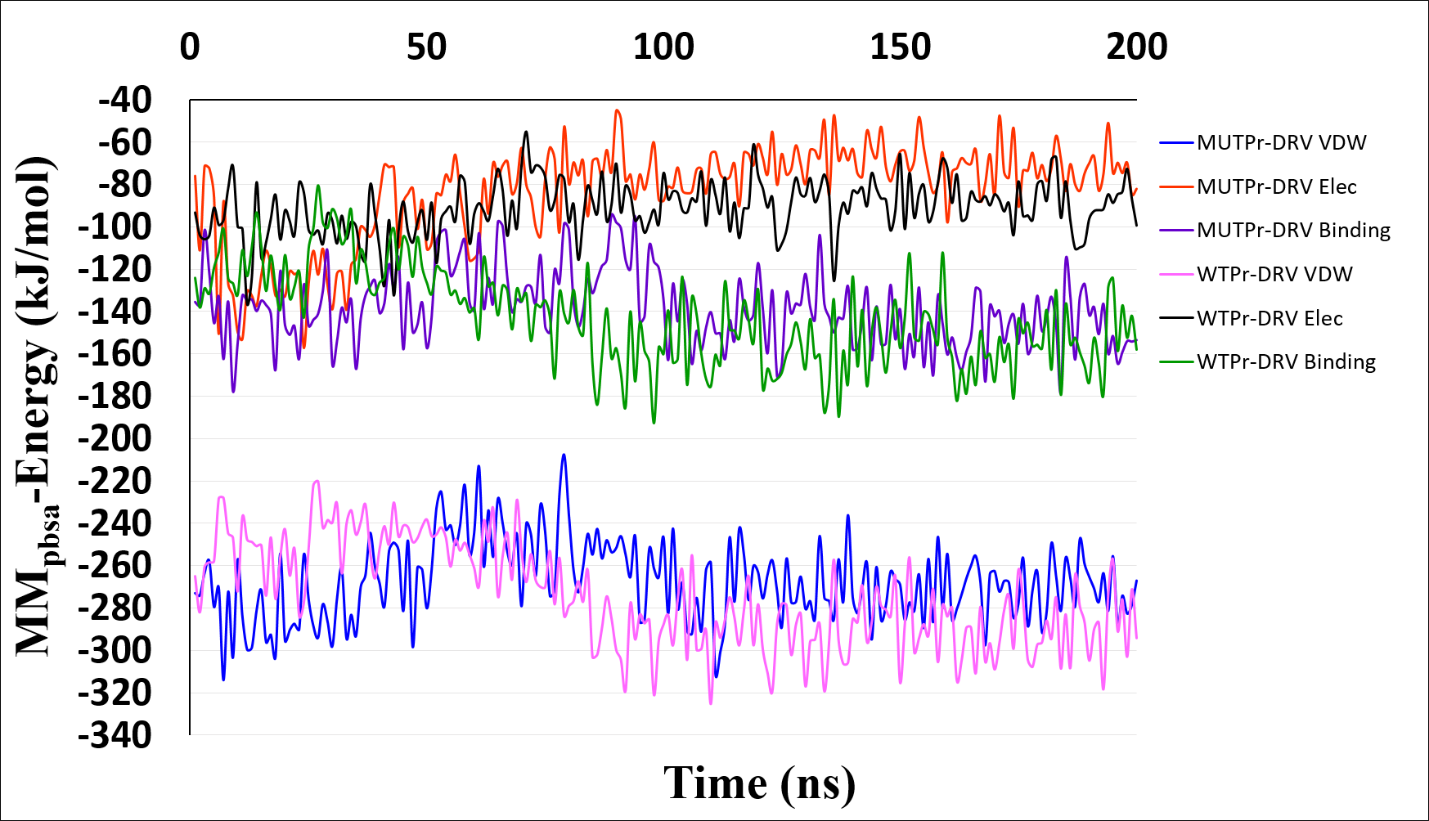


Figure S8) The plot show DRV van der Waals, electrostatic and total binding energy (enthalpy of MM_pbsa_) for both WT-Pr and MUT-Pr (during 200 ns).


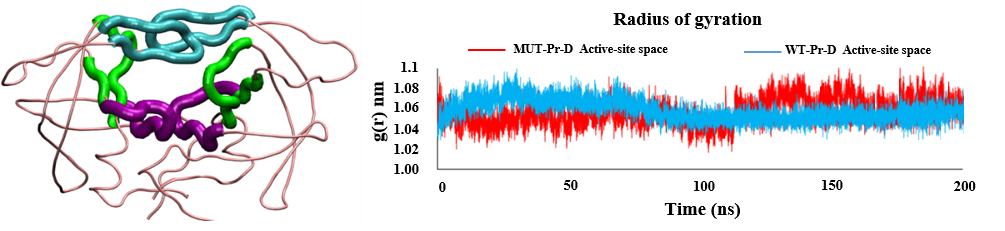


Fig. S9. Left) Thick tube shapes show the residues around the active site, which was used to calculate the radius of gyration; Right) The radius of gyration based on the backbone atoms of mentioned residues that are around the active site space.

Table S1) Decomposition of binding energy on a per-residue basis

| **∆G_MMPBSA** | **∆G_nonpol-solv_** | **∆G_pol-solv_** | **∆E_MM**  **(VDW + elec)** | **Residue** | |
| --- | --- | --- | --- | --- | --- |
| **0.81 ± 0.22** | **-0.23 ± 0.01** | **-0.15 ± 0.26** | **1.19 ± 0.11** | **WT-Pr** | **R8** |
| **6.75 ± 0.30** | **-0.18 ± 0.01** | **3.34 ± 0.34** | **3.58 ± 0.09** | **MUT-Pr** |  |
| **5.94** | **0.05** | **3.49** | **2.39** | **Diff** |  |
| **-8.36 ± 0.17** | **-0.58 ± 0.02** | **4.48 ± 0.09** | **-12.27 ± 0.21** | **WT-Pr** | **I50** |
| **-5.45 ± 0.20** | **-0.39 ± 0.01** | **1.28 ± 0.11** | **-6.35 ± 0.27** | **MUT-Pr** |  |
| **2.91** | **0.19** | **-3.2** | **5.92** | **Diff** |  |
| **-10.15 ± 0.14** | **-0.70 ± 0.02** | **-0.03 ± 0.00** | **-9.42 ± 0.13** | **WT-Pr** | **I84** |
| **-4.98 ± 0.13** | **-0.33 ± 0.01** | **-0.15 ± 0.01** | **-4.51 ± 0.13** | **MUT-Pr** |  |
| **5.17** | **0.37** | **-0.12** | **4.91** | **Diff** |  |
| **-3.66 ± 0.12** | **-0.16 ± 0.01** | **-2.00 ± 0.07** | **-1.49 ± 0.11** | **WT-Pr** | **D25’** |
| **13.92 ± 0.51** | **-0.24 ± 0.01** | **37.24 ± 0.53** | **-23.07 ± 0.19** | **MUT-Pr** |  |
| **17.58** | **-0.08** | **39.24** | **-21.58** | **Diff** |  |
| **-6.68 ± 0.12** | **-0.61 ± 0.02** | **2.09 ± 0.05** | **-8.16 ± 0.13** | **WT-Pr** | **A28’** |
| **-4.67 ± 0.13** | **-0.47 ± 0.01** | **1.51 ± 0.06** | **-5.71 ± 0.12** | **MUT-Pr** |  |
| **2.01** | **0.14** | **-0.58** | **2.45** | **Diff** |  |

All the energies are in KJ/mol. ∆E_MM (molecular mechanic energy); the ∆E_vdw_  (van der Waals interaction energy); ∆E_electrostatic_ (Electrostatic interaction energy in gas phase); ∆G_polar-solvation_ (Polar solvation energy); ∆G_SASA_ (Non-Polar solvation energy).
